# Genetics Variants in the Epoxygenase Pathway of Arachidonic Metabolism Are Associated with Eicosanoids Levels and the Risk of Diabetic Nephropathy

**DOI:** 10.3390/jcm10173980

**Published:** 2021-09-02

**Authors:** Sonia Mota-Zamorano, Nicolás R. Robles, Luz M. González, José M. Valdivielso, Juan Lopez-Gomez, Bárbara Cancho, Guadalupe García-Pino, Guillermo Gervasini

**Affiliations:** 1 Department of Medical and Surgical Therapeutics, Division of Pharmacology, Medical School, University of Extremadura, 06071 Badajoz, Spain; smotazamorano@gmail.com (S.M.-Z.); lmgonzalezgarcia89@gmail.com (L.M.G.); 2 Service of Nephrology, Badajoz University Hospital, 06071 Badajoz, Spain; nrrobles@unex.es (N.R.R.); bcancho@yahoo.es (B.C.); 3 Vascular and Renal Translational Research Group, UDETMA, ISCIII REDinREN, IRBLleida, 25198 Lleida, Spain; valdivielso@irblleida.cat; 4 Service of Clinical Analyses, Badajoz University Hospital, 06071 Badajoz, Spain; lopezhospi@yahoo.es; 5 Zafra Hospital, 8105 Zafra, Spain; guadalupegpino@hotmail.com

**Keywords:** chronic kidney disease, diabetic kidney disease, diabetes mellitus, CYP4F2

## Abstract

Genes in the epoxygenase pathway of arachidonic acid metabolism leading to vasoactive eicosanoids, mainly 20-hydroxyeicosatetraenoic (20-HETE) and epoxyeicosatrienoic (EETs) acids, have been related to glucose-induced renal damage in preclinical reports. We genotyped 1088 diabetic kidney disease (DKD) patients and controls for seven polymorphisms in five genes (*CYP2C8*, *CYP2J2*, *CYP4F2*, *CYP4A11*, and *EPHX2*) along this metabolic route and evaluated their effect on DKD risk, clinical outcomes, and the plasma/urine levels of eicosanoids measured by LC/MS/MS and immunoenzymatic assays. The *CYP4F2* 433M variant allele was associated with lower incidence of DKD (OR = 0.65 (0.48–0.90), *p* = 0.008), whilst the CYP2C8*3/*3 genotype was related to increased risk (OR = 3.21 (1.05–9.87), *p* = 0.036). Patients carrying the 433M allele also showed lower eGFR [median and interquartile range vs. wildtype carriers: 30.8 (19.8) and 33.0 (23.2) mL/min/1.73 m^2^, *p* = 0.037). Finally, the 433VM/MM variant genotypes were associated with lower urinary levels of 20-HETE compared with 433VV (3.14 (0.86) vs. 8.45 (3.69) ng/mg Creatinine, *p* = 0.024). Our results indicate that the *CYP4F2* V433M polymorphism, by decreasing 20-HETE levels, may play an important role in DKD.

## 1. Introduction

The incidence of chronic kidney disease (CKD) has greatly increased in recent years to become a global healthcare issue. CKD is suffered by 10% of the world’s population and it will be one of the most frequent causes of death by 2040 [1]. The greatest contributor to CKD is diabetic kidney disease (DKD), which refers to pathologic structural and functional changes seen in the kidneys of patients with diabetes mellitus (DM). At least 40% of patients with type 2 DM will develop DKD [2], which accounts for over 50% of individuals who require a dialysis treatment and/or renal transplant in some parts of the world [3].

Proteinuria has traditionally been considered the hallmark of DKD [4]. Other indicators include decreased glomerular filtration rate (GFR) or elevated arterial blood pressure [5], which altogether can help the nephrologist to establish a diagnosis that can later be histologically confirmed. However, it is now known that there is a significant proportion of subjects with DKD who, in spite of decreased GFR, do not have elevated concentrations of proteins in urine [6,7]. Therefore, the necessity of new, universal, and more specific biomarkers of DKD has been repeatedly pointed out [8,9].

Several in vitro and animal studies indicate that the cytochrome P450 (CYP450)-mediated epoxygenase pathway of arachidonic acid (AA) (Figure S1) plays a major role in the onset of diabetes and its renal complications [10,11,12]. In addition, our group has reported that the levels of eicosanoids generated in this metabolic route, of which EETs and 20-HETE are the most important, are associated with the risk of DKD and modulate parameters of renal function in these patients [13].

Genetics is receiving increasing attention regarding its role in DKD [14]. In this regard, we have previously shown that the onset of post-transplant diabetes mellitus in renal transplant recipients is dependent on the presence of variants in genes expressed in the kidney that participate in the epoxygenase pathway [15]. In addition, these polymorphisms have also been related to the development of renal injury [16,17], and cardiovascular (CV) disease [18], whose risk is greatly elevated in DKD patients. However, in spite of this background, the influence of genetic polymorphisms in the AA epoxygenase route on DKD remains untested.

Our aim was to determine whether seven functional, common polymorphisms in the five genes mediating this metabolic pathway (*CYP2J2*, *CYP2C8*, *CYP4A11*, *CYP4F2*, and *EPHX2*) are involved in the incidence of DKD and/or clinical outcomes in these patients. In search of a mechanistic explanation for the putative SNP-disease associations, we also aimed to establish whether these variants could modulate the levels of vasoactive eicosanoids measured in plasma and/or urine.

## 2. Patients and Methods

### 2.1. Study Subjects

A total of 1088 subjects were included in this study, 430 patients with DKD (stage 3 or higher) and 658 individuals with normal renal function. Participants were obtained from the Nephrology Service and the Hypertension Unit of the Badajoz University Hospital, where they were recruited over a four-year period (2017–2020), and from the NEFRONA repository, which archives biological samples that were collected in a former multicenter study of CV morbidity and mortality in Spanish CKD patients and healthy subjects [19].

Patients were over 18 years of age and had had type 2 diabetes (fasting glucose > 126 mg/dL or non-fasting glucose > 200 mg/dL) prior to kidney damage (eGFR < 60 mL/min and albuminuria). Overt albuminuria was considered when albumin excreted in urine over 24 h was higher than 300 mg. Microalbuminuria was defined as 30–300 mg albumin in 24-h urine. DKD was diagnosed histologically or attending to clinical criteria, i.e., the presence of both albuminuria and retinopathy after excluding other probable causes. A biopsy was carried out for confirmation of the DKD diagnosis when proteinuria was higher than 1 g/24 h. Prognostic stratification of patients was conducted with the KDIGO classification and the CONSORTIUM-CKD equation (Kidney Risk Failure; www.kidneyriskfailure.org, accessed on 15 April 2021). A modification of the Diet in Renal Disease (MDRD) equation was used to estimate renal function, as it has been shown to give reliable estimates of the GFR slope in patients with CKD [20]. CV risk was defined as the likelihood of experiencing a fatal or non-fatal CV event in the 4-year follow-up (54 months). CV events included acute myocardial infarction, acute coronary syndrome, coronary catheterization requiring angioplasty, coronary bypass, typical angina with positive stress tests, sudden death, cerebrovascular accident, peripheral arterial disease, and aortic aneurisma. Age- and sex-matched controls with eGFR > 60 mL/min/1.73 m^2^ were recruited from the Badajoz University Hospital or from primary care centers throughout the country (in the case of samples obtained from the NEFRONA cohort). Exclusion criteria for participation in the study included previous history of any CV event, transplantation of any organ, carotid artery surgery, active infection, pregnancy, or life expectancy below one year.

This study was approved by the Ethics Committee of the Badajoz University Hospital, and it was carried out in accordance with the Declaration of Helsinki and its subsequent revisions. All subjects gave written informed consent for their participation.

### 2.2. Genetic Analysis

Twelve-mL blood samples were extracted from the participants and DNA was subsequently purified by a standard phenol-chloroform extraction method. In the case of the subjects recruited in the former NEFRONA study, DNA was obtained from biological samples stored at the REDinREN biobank [21] with QIAamp DNA Blood kits following the manufacturer’s instructions. Seven SNPs in the five genes of the epoxygenase pathway in AA metabolism were searched for, namely CYP2J2*7 (rs890293), CYP2C8*3 (rs10509681), *CYP4F2* V433M (rs2108622), *CYP4A11* F434S (rs1126742), *EPHX2* 3′UTR A>G (rs1042032), *EPHX2* R287Q (rs751141), and *EPHX2* K55R (rs41507953). Genotyping was carried out by real-time PCR using commercial TaqMan^®^ SNP genotype assays from Thermofisher Scientific (Rockford, Il, USA). These SNPs were selected based on previous reports stating their influence on clinical outcomes [18,22,23,24,25,26].

### 2.3. Determination of Plasma and Urinary Levels of Eicosanoids

Vasoactive eicosanoids, namely 20-HETE, 14,15-DHET, and 11,12-DHET, could be measured in a group of 334 patients and controls of whom plasma samples were available. Since 20-HETE urinary excretion has been suggested to be a marker of human disease [13,27,28], we also measured concentrations of this metabolite in urine after correcting for renal function (ng 20-HETE/mg Creatinine (Cr)). DHETs, the direct product of EETs biotransformation by *EPHX2*, are far easier to quantify than their analogous EETs, which disappear rapidly from the biological matrix, and hence they were used as surrogates as described elsewhere [25,29]. For plasma determinations, we processed 0.5-mL aliquots by solid-phase extraction in Hypersep Retain Pep 60 mg 3 mL S columns (Thermofisher Scientific, Waltham, MA, USA). Quantification of the AA metabolites was carried out by mass spectrometry coupled to liquid chromatography (LC/MS/MS) using a UHPLC 1290 system with a 6460 Jet Stream triple quadrupole mass detector (Agilent Technologies, Santa Clara, CA, USA), as previously described [30]. Glucuronidated and free 20-HETE concentrations were also determined in urine by a beta-glucuronidase competitive immunoenzymatic assay kit (Abcam, Cambridge, UK). Inter- and intra-assay variation tests results were below 15 and 10%, respectively. A previous digestion was performed with beta-glucuronidase to allow the identification of conjugated 20-HETE. In brief, samples and standards were diluted and the conjugate was added to the wells as per the manufacturer’s instructions. Following incubation and washing procedures, TMB substrate was added before a final incubation at room temperature. 2N sulphuric acid was used to stop the reaction and the plates were read at 450 nm with a Biotek ELx808 plate reader (Biotek Instruments Inc., Winooski, VT, USA).

### 2.4. Statistical Analyses

Pearson’s X^2^ or Fisher’s exact were used to identify differences between categorical variables. Differences between quantitative variables were evaluated with the Student’s t/ANOVA or Mann–Whitney/Kruskal–Wallis tests, as appropriate. Binary logistic regression was carried out to evaluate the impact of the SNPs on the risk of DKD controlling for confounding variables as formerly described [13]. Multivariate linear regression analyses were performed to analyze the influence of genetics on renal parameters. Albumin-to-creatinine ratios (ACR) and eGFR values were log-transformed before their inclusion in the models. Relevant covariates used in the models included sex, age, weight, CKD stage, hyperlipidemia, and hypertension. The association of the different genotypes with CV event-free survival was assessed by Kaplan–Meier curves and the results were compared with the log-rank test. Patients were followed up until the earliest CV event, death, or end of the study. Statistical analyses were performed with the IBM SPSS statistics 22 (Chicago, IL, USA).

## 3. Results

Table 1 summarizes demographic and clinical characteristics of the study participants. All the SNPs studied were in Hardy–Weinberg equilibrium and showed frequencies that were similar to those reported in 1000 genomes (www.internationalgenome.org, accessed on 15 April 2021) for the Iberian population in Spain (Table 2). Genotyping was successful in 98.6% of the samples.

### 3.1. Case-Control Study

We compared the distribution of the studied polymorphisms between the 430 patients with stage 3 or higher DKD and 658 individuals with normal renal function (eGFR > 60 mL/min/1.73 m^2^). After adjusting for confounding variables, carriers of the *CYP4F2* 433M variant allele were found to be at lower risk of developing DKD (OR = 0.65 (0.48–0.90), *p* = 0.008, Table 3). The other significant association was observed for CYP2C8*3. Carriers of the homozygous variant genotype showed increased susceptibility to the disease (OR = 3.21 (1.05–9.87), *p* = 0.036, Table 3). None of the other SNPs showed a relevant relation to DKD risk.

A small subset (*n* = 65) of the control subjects without renal impairment had diabetes. The comparison of these individuals with the patient group resulted again in the *CYP4F2* 433 VM/MM genotypes being associated with a lower risk of DKD (OR = 0.42 (0.22–0.80), *p* = 0.006; Table 4). In addition, for this subgroup, the *EPHX2* 55KR/RR variant genotypes were also inversely related to DKD risk (OR = 0.39 (0.21–0.76), *p* = 0.006, Table 4). No significant results were obtained with the recessive model of inheritance (not shown in Table 4).

### 3.2. Effect of SNPs on Renal Parameters of DKD Patients

First, we examined the effect of the studied SNPs on the renal function of the DKD patients who were not on dialysis at the time of the study. After controlling for meaningful covariates, carriers of the 433M variant allele displayed significantly lower eGFR than wild-type subjects (median (and interquartile range) for VM/MM vs. VV genotypes were, respectively, 30.8 (19.8) vs. 33.0 (23.2) mL/min/1.73 m^2^, *p* = 0.037, Table 5). In contrast, the analysis of ACR values did not reveal any relevant associations (Table 5).

### 3.3. Analysis of Cardiovascular Risk in the DKD Cohort

The median follow-up time of the DKD patients was 47 months (range 7–54). In this period, a total of 92 patients (21.4%) experienced CV events. Kaplan–Meier analyses did not show any significant associations between the studied SNPs and cumulative CV event-free survival. Figure S2 depicts survival curves and log-rank test results for all the SNPs.

### 3.4. Association between Eicosanoids Levels and Epoxygenases Polymorphisms

Concentrations of vasoactive eicosanoids were assessed in a subset of 132 DKD patients and 202 controls. In a previous study [13], we had established that these concentrations were different between DKD patients and non-diabetic subjects, and therefore, we analyzed the influence of genetics in both groups separately. No associations were observed in the control group (Table S1). In the patient group, however, carriers of the *CYP4F2* 433M variant allele displayed significantly lower urinary levels of 20-HETE corrected by creatinine values than wild-type patients did. Mean (and standard error) values of 433M carriers vs. non carriers were, respectively, 3.14 (0.86) vs. 8.45 (3.69) ng/mg Cr, *p* = 0.024 (Table 6).

## 4. Discussion

In the last years, we and others have pointed out the importance of vasoactive eicosanoids and their encoding genes in the cardiorenal function of renal patients and kidney transplant recipients [13,18,26,31,32,33,34]. Furthermore, preclinical reports indicate that these compounds are closely related (directly in the case of 20-HETE or inversely in the case of EETs) to the damage induced by hyperglycemia on renal cells [11,12,35,36]. Altogether, these data support the hypothesis tested in the present work, namely that genetic variants in the AA epoxygenase metabolism may be relevant for DKD risk and outcomes.

The results of the risk analysis showed that carriers of a valine (V)-to-methionine (M) substitution in amino acid 433 of CYP4F2 (CYP4F2*3, *G1347A*) had increased susceptibility to DKD. CYP4F2 is responsible for the synthesis of 20-HETE [37] and there is evidence in cell cultures and animal models indicating that 20-HETE plays a key role in the etiopathology of type 2 DM through the impairment of insulin signaling [38,39]. Moreover, in vitro reports show that 20-HETE is an important mediator of hyperglycemia-mediated kidney injury through several mechanisms [11,12,40], and CYP4 inhibitors and 20-HETE antagonists have been suggested to hold an important therapeutic potential in the treatment of diabetic complications [11]. Moving on to clinical studies, we recently reported that DKD patients had reduced excretion of 20-HETE in urine in comparison with control subjects, suggesting an accumulation of this eicosanoid in tissues where CYP4F2 is highly expressed such as the kidney [13]. Since the 433M allele has been shown to significantly decrease 20-HETE synthesis in vitro [41,42], we hypothesized that the decreased DKD risk observed in carriers of this variant may obey to a deactivation of the aforementioned pathological mechanisms mediated by 20-HETE. Indeed, our analysis of samples obtained from DKD patients confirmed this hypothesis, as carriers of the variant M-allele had lower levels of 20-HETE after correcting for renal function. This is the first time to our knowledge that the consequences of SNPs in the AA epoxygenase pathway have been tested in vivo.

On the other hand, in a previous study on renal transplant recipients, we had observed that V433M SNP was associated with the onset of post-transplant diabetes mellitus (PTDM) [15]. Although it is remarkable that the same SNP was also pinpointed in this cohort, the 433M allele was related to an increased risk of PTDM in this case. Causes for this discrepancy may obey the existence of different mechanisms in the etiology of PTDM and DKD. Thus, 20-HETE has been suggested to be able to exert opposite functions in kidney homeostasis depending on the cell type that produces and/or targets this eicosanoid [40].

Interestingly, the analysis of the patient cohort showed that the same *CYP4F2* V433M SNP that decreased the susceptibility to DKD was also related to lower eGFR values. It is tempting to speculate that these two findings might be related. Carriers of the 433M variant (at lower risk) would theoretically produce less 20-HETE resulting in a reduction of the vasoconstrictor activity in renal tissue [43]. In turn, this would alleviate glomerular capillary pressure causing the observed reduction in filtration. This protective mechanism would be similar to that shown by drugs used in CKD, such as RAAS blockers [44] or SGLT2 inhibitors [45]. These medications lower single nephron GFR and reduce the proximal tubular workload, therefore, protecting tubules that may already be compromised by decreased oxygen availability because of the capillary rarefaction characteristics of CKD [46].

The other SNP that was associated with DKD risk was CYP2C8*3, as homozygous carriers displayed higher susceptibility to the disease. CYP2C8 is the main enzyme responsible for EETs synthesis in the kidney and it is known that the *3 variant significantly reduces the biotransformation of AA to these eicosanoids both in vitro [47] and in vivo [48]. EETs are basically renoprotective compounds [49,50] that, amongst other functions, display vasodilator and anti-inflammatory properties [33,51]. Therefore, it is likely that carriers of the *3/*3 genotype have less endogenous resources to counteract hyperglycemia-induced damage in renal cells. We could not confirm a difference in plasma levels of DHETs between *CYP2C8* genotypes, although a peripheral measurement of these levels might not reflect the concentrations in renal tissue.

An interesting finding was that *CYP4F2* 433M and *EPHX2* 55R allelic variants were inversely related to DKD risk when only a subset of diabetic subjects with normal renal function was considered as the control group. The fact that the V433M SNP, which reduces 20-HETE synthesis, still shows a relevant effect when diabetes is taken out of the equation and only filtration is considered, indicates that 20-HETE must be an important factor for renal function also in mechanisms independent from glucose actions. In this regard, Gangadhariah et al. reported that hypertension is a major contributor to the deleterious actions of 20-HETE in DKD [40]. On the other hand, the protective effect of the *EPHX2* 55R variant was a surprising result, as this polymorphism has been associated with increased soluble epoxyhydrolase activity (sEH) [48,52], which would presumably lead to a reduction in the concentration of renoprotective EETs. Therefore, we do not have a plausible explanation for the OR value observed for this SNP other than there could be an interaction with other functional *EPHX2* SNPs leading to a different net effect on EETs levels, as it has been recently reported [48]. In any case, and in spite of its effect on sEH activity, the impact of this the *EPHX2* K55R SNP on EETs levels has been shown to be negligible or small at best [48,53].

These genes mediating AA metabolism have long been claimed to affect cardiovascular function [54]. We have previously reported that both CYP2J2*7 and CYP2C8*3 are related to the occurrence of CV events in renal transplant recipients without a previous CV history [18]. In contrast, in the present DKD cohort, we could not identify any relevant CV associations, which could be due to phenotypic differences between the study populations (renal patients vs. renal recipients). Indeed, even though both populations are groups with a high incidence of CV disease, the occurrence of CV events in the DKD cohort was much more frequent than in the previous transplant cohort (21.4 vs. 11.0 %), in spite of the much longer follow-up of the latter.

A limitation of this study was that the sample size was relatively small, particularly in the subgroup of diabetic individuals with normal renal function. In addition, 20-HETE/Cr determinations could not be carried out in subjects on dialysis (as there was no urine available). Finally, the observed association between 433M and low 20-HETE/Cr ratios in urine was absent in the control group. On this last aspect, we would need a deeper knowledge of the true role of 20-HETE in the cardiorenal function to explain why the 433M variant did not show a significant effect in controls. Other factors present in diabetic patients with renal impairment must contribute to the observed differences. For instance, an overexpression of CYP4F2 in renal tissue producing more 20-HETE in DKD patients [12,55] could make mild differences due to genetic variants being easier to detect than in non-diabetic individuals.

Currently, the development of new treatments for DKD is stalling for a variety of reasons, of which the lack of new predictive and prognostic biomarkers for a more accurate patient stratification is particularly important. In the present work, we have tested the hypothesis that genetic variants in the epoxygenase pathway of AA metabolism may be relevant in this regard. Our results show that the V433M SNP in the *CYP4F2* gene, which is responsible for the synthesis of 20-HETE, a major mediator in hyperglycemia-induced renal cell damage, is associated with a decreased risk of DKD and low eGFR values in these patients, which suggests this could be a useful marker in the diagnosis and follow-up of DKD patients. A reduction in the synthesis of 20-HETE could be the mechanism explaining the observed associations. Our results add to the existing body of recent evidence pointing to this metabolic route as a promising therapeutic drug target in DKD.

## Figures and Tables

**Table 1:** Descriptive and clinical characteristics of the population of study. Values shown are count (and percentage) or median (and interquartile range).

	Control	CKD 3	CKD 4–5	CKD 5D	Total	*p*-Value Control vs. CKD	*p*-Value between CKD Groups
N	658	161	140	129	1088		
Age (yrs)	57 (17)	65 (13)	63.5 (17)	65 (19)	59 (18)	4.51 × 10^−21^	0.123
Males (%)	359 (54.6)	112 (69.6)	91 (65.0)	88 (68.2)	650 (59.7)	1.0 × 10^−5^	0.691
Weight (kg)	76.8 (21)	80 (22.6)	79 (18.1)	72.5 (21.5)	77 (20.40)	0.060	0.001
Hypertension	297 (45.1)	154 (95.7)	136 (97.1)	123 (95.3)	710 (65.3)	5.50 × 10^−79^	0.714
Hyperlipidemia	199 (30.2)	121 (75.2)	109 (77.9)	83 (64.3)	512 (47.1)	4.08 × 10^−44^	0.031
Creatinine (mg/dL)	0.7 (0.5)	0.6 (0.4)	0.5 (0.1)	-	0.7 (0.4)	0.017	0.030
Albumin/Creatinine (mg/g)	7.2 (27.8)	152.4 (583.3)	283.6 (1052.1)	-	24.3 (173.5)	7.24 × 10^−31^	0.046
Cardiovascular events	9 (1.8)	28 (17.4)	25 (17.9)	39 (30.2)	101 (10.8)	5.05 × 10^−24^	0.014
eGFR (mL/min/1.73 m²)						5.91 × 10^−253^	0.285
<60	-	159 (98.8)	140 (100.0)	-	299 (31.2)		
>60	658 (100.0)	2 (1.2)	-	-	658 (68.8)		

CKD, chronic kidney disease; CKD-5D, dialysis; eGFR, estimated glomerular filtration rate.

**Table 2:** Summary of genotyping results.

Polymorphism	rs Number	Alleles	Missing (%)	HWE	MAF (%)	MAF IBS (%)	^a^*p*-Value
CYP2C8 *1/*3	rs10509681	A/G	1.3	1.0	14.8	15.0	0.965
CYP2J2 *1/*7	rs890293	G/T	1.0	0.387	5.7	6.1	0.729
*CYP4F2 V433M*	rs2108622	C/T	1.7	0.057	36.9	35.5	0.475
*CYP4A11 F433S*	rs1126742	A/G	2.1	0.400	14.9	13.6	0.806
*EPHX2 R287Q*	rs751141	G/A	1.0	0.424	6.1	7.5	0.806
*EPHX2 3′UTR*	rs1042032	A/G	1.6	0.358	24.0	18.7	0.548
*EPHX2 K55R*	rs41507953	A/G	1.0	0.730	9.7	7.0	0.420

HWE, Hardy-Weinberg equilibrium; MAF, minor allele frequency; IBS, Iberian populations in Spain. ^a^ *p*-value for the difference between minor allele frequencies.

**Table 3:** Adjusted analysis of the association of polymorphisms in the epoxygenase pathway of arachidonic acid metabolism with the risk of diabetic kidney disease (DKD). Results for the dominant and recessive models are shown.

						Dominant Model			Recessive Model		
Polymorphism	Genotype	Controls	%	DKD	%	OR	CI	*p*	OR	CI	*p*
CYP2C8 *1/*3	*1/*1	457	70.3	323	76.2	0.81	(0.57–1.14)	0.225	3.21	(1.05–9.87)	0.036
	*1/*3	182	28	89	21						
	*3/*3	11	1.7	12	2.8						
CYP2J2*7 *1/*7	*1/*1	579	89.1	380	89	1.01	(0.60–1.68)	0.975	-	-	-
	*1/*7	66	10.2	47	11						
	*7/*7	5	0.8	0	0						
*CYP4F2 V433M*	V/V	249	38.6	192	45.2	0.65	(0.48–0.91)	0.008	0.7	(0.46–1.07)	0.097
	V/M	299	46.4	170	40						
	M/M	97	15	63	14.8						
*CYP4A11 F434S*	F/F	463	71.9	311	73.9	0.8	(0.57–1.13)	0.198	1.54	(0.61–3.88)	0.354
	F/S	167	25.9	97	23						
	S/S	14	2.2	13	3.1						
*EPHX2 R287Q*	R/R	570	87.7	377	88.3	0.75	(0.47–1.18)	0.216	0.79	(0.04–1.19)	0.22
	R/Q	79	12.2	49	11.5						
	Q/Q	1	0.2	1	0.2						
*EPHX2 3′UTR A>G*	A/A	382	59	242	57.1	0.97	(0.71–1.33)	0.845	1.05	(0.57–1.95)	0.87
	A/G	226	34.9	154	36.3						
	G/G	39	6	28	6.6						
*EPHX2 K55R*	K/K	532	81.7	346	81.2	0.82	(0.56–1.21)	0.319	1.32	(0.31–5.69)	0.709
	K/R	113	17.4	75	17.6						
	R/R	6	0.9	5	1.2						

OR, odds ratio; CI, 95% confidence intervals.

**Table 4:** Risk analysis considering the 430 diabetic kidney disease (DKD) patients and a subgroup of diabetic individuals with normal renal function.

Polymorphism	Genotype	Diabetics without DKD (*n* = 65)	%	DKD (*n* = 430)	%	OR	CI	*p*-Value
CYP2C8 *1/*3	*1/*1	47	72.3	323	76.2	Ref.		
	*1/*3-*3/*3	18	27.7	101	23.8	0.95	(0.49–1.85)	0.883
CYP2J2 *1/*7	*1/*1	59	90.8	380	89	Ref.		
	*1/*7-*7/*7	6	9.2	47	11	1.19	(0.45–3.15)	0.724
*CYP4F2 V433M*	VV	18	27.7	192	45.2	Ref.		
	VM-MM	47	72.3	233	54.8	0.42	(0.22–0.80)	0.005
*CYP4A11 F433S*	FF	44	67.7	311	73.9	Ref.		
	FS-SS	21	32.3	110	26.1	0.39	(0.39–1.37)	0.330
*EPHX2 R287Q*	RR	56	86.2	377	88.3	Ref.		
	RQ-QQ	9	13.8	50	11.7	0.77	(0.33–1.81)	0.561
*EPHX2 3′UTR (A/G)*	AA	37	56.9	242	57.1	Ref.		
	AG-GG	28	43.1	182	42.9	0.92	(0.51–1.65)	0.773
*EPHX2 K55R*	KK	44	67.7	346	81.2	Ref.		
	KR-RR	21	32.3	80	18.8	0.39	(0.21–0.76)	0.006

OR, odds ratio; CI, 95% confidence intervals; Ref, reference. V, valine; M, methionine; F, phenylalanine; S, serine; R, arginine; Q, glutamine; K, lysine.

**Table 5:** Adjusted analysis for determining associations of the different genotypes with renal parameters of patients with diabetic kidney disease.

		eGFR (mL/min/1.73 m^2^)			ACR (mg/g)		
Polymorphism	Genotype	Median	IQR	*p*-Value	Median	IQR	*p*-Value
CYP2C8 *1/*3	*1/*1	32.14	22	0.208	195.12	853.21	0.787
	*1/*3-*3/*3	30.61	22		199.05	425.67	
CYP2J2 *1/*7	*1/*1	31.31	22	0.247	199.56	646.66	0.231
	*1/*7-*7/*7	34.43	23		131.0	1261.20	
*CYP4F2 V433M*	VV	33.0	23	0.037	165.28	911.65	0.982
	VM-MM	30.80	20		231.95	679.33	
*CYP4A11 F433S*	FF	31.24	22	0.823	186.94	659.79	0.387
	FS-SS	33.0	22		228.60	855.30	
*EPHX2 R287Q*	RR	32.47	22	0.286	199.56	828.48	0.861
	RQ-QQ	27.90	23		131.0	489.27	
*EPHX2 3′UTR (A/G)*	AA	30.41	24	0.284	152.43	597.33	0.435
	AG-GG	33.0	21		299.12	835.90	
*EPHX2 K55R*	KK	30.90	22	0.051	167.74	653.37	0.136
	KR-RR	35.85	23		386.0	846.14	

eGFR, estimated glomerular filtration rate; ACR, albumin-to-creatinine ratio; IQR, interquartile range.

**Table 6:** Influence of polymorphisms in the epoxygenase pathway on the plasma and urinary levels of vasoactive eicosanoids shown by patients with diabetic kidney disease.

		14,15-DHET (ng/L)		11,12-DHET (ng/L)		20-HETE (ng/L)		20-HETE ng/mg Cr	
Polymorphism	Genotype	Mean	SE	Mean	SE	Mean	SE	Mean	SE
CYP2C8 *1/*3	*1/*1	397.35	26.68	237.65	18.79	306.96	19.89	6.09	2.41
	*1/*3 *-* 3*/*3	409.80	54.44	238.86	42.53	322.93	57.51	4.76	1.74
CYP2J2 *1/*7	*1/*1	409.40	25.91	239.56	19.04	315.41	22.4	5.85	2.03
	*1/*7 *-* *7/*7	288.78	34.69	206.75	35.48	268.22	57.32	4.92	1.55
*CYP4F2 V433M*	VV	393.73	36.59	226.16	26.39	309.75	35.58	8.45	3.69
	VM-MM	401.42	31.91	246.15	23.67	310.68	24.43	*3.14	0.86
*CYP4A11 F433S*	FF	409.32	26.88	246.43	18.88	306.21	19.85	6.0	2.53
	FS-SS	370.85	50.07	212.58	39.33	319.82	53.04	5.19	1.63
*EPHX2 R287Q*	RR	396.03	23.93	233.66	17.15	312.08	22.32	5.8	2.10
	RQ-QQ	421.75	133.57	275.29	117.82	286.75	42.62	5.76	3.48
*EPHX2 3′UTR (A/G)*	A/A	373.74	18.8	219.20	13.14	308.3	21.07	6.74	2.77
	A/G-G/G	446.16	61.15	269.47	44.72	314.16	47.0	3.78	0.95
*EPHX2 K55R*	KK	392.65	24.35	231.07	18.47	322.41	23.58	6.02	2.21
	KR-RR	439.07	93.22	272.93	59.05	222.64	28.36	4.41	1.73

DHET, dihydroxyeicosatrienoic acid; HETE, hydroxyeicosatetraenoic acid; SE, standard error; * *p* < 0.05.

## Data Availability

The data that support the findings of this study are available on request from the corresponding author.

## Supplementary Materials

The following are available online at https://www.mdpi.com/article/10.3390/jcm10173980/s1, Figure S1: Epoxygenase pathway of arachidonic acid metabolism, Figure S2: Cumulative event-free survival for patients with diabetic kidney disease according to the different genotypes considered, Table S1. Influence of polymorphisms in the epoxygenase pathway on the plasma and urinary levels of vasoactive eicosanoids shown by subjects with normal renal function.

## Abbreviations

20-HETE, 20-hydroxyeicosatetraenoic acid; ACR, albumin-to-creatinine ratio; CKD, chronic kidney disease; DHETs, dihydroxyeicosatrienoic acids; DKD, diabetic kidney disease; EETs, epoxyeicosatrienoic acids; eGFR, estimated glomerular filtration rate; IQR, interquartile range; LC/MS/MS, mass spectrometry coupled to liquid chromatography; MDRD, modification of diet in renal disease; SNP, single nucleotide polymorphism.
