# Supplementary material for: Genetics Variants in the Epoxygenase Pathway of Arachidonic Metabolism Are Associated with Eicosanoids Levels and the Risk of Diabetic Nephropathy
Source: J Clin Med. 2021 Sep 2;10(17):3980. doi: 10.3390/jcm10173980 (PMC8432556; doi:10.3390/jcm10173980)

Supplementary materials

Table S1. Influence of polymorphisms in the epoxygenase pathway on the plasma and urinary levels of vasoactive eicosanoids shown by subjects with normal renal function.

		**14,15-DHET (ng/L)**		11,12-DHET (ng/L)		20-HETE (ng/L)		20-HETE ng/ mg Cr	
**Polymorphism**	**Genotype**	**Mean**	**SE**	Mean	SE	Mean	SE	Mean	SE
*CYP2C8 *1/*3*	**1/*1*	526.55	27.02	279.87	12.49	309.72	16.33	9.75	02.04
	**1/*3 y 3*/*3*	610.84	62.1	296.37	19.28	314.61	20.94	15.87	4.73
*CYP2J2 *1/*7*	**1/*1*	539.12	26.77	281.24	11.22	311.2	14.28	11.47	2.12
	**1/*7-*7/*7*	635.93	91.24	311.71	28.05	308.93	31.16	9.97	3.98
CYP4F2 V433M	VV	563.67	42.78	278.72	15.31	294.72	19.01	12.58	3.51
	VM-MM	538.35	32.25	287.75	14.26	321.57	17.99	10.48	2.27
CYP4A11 F433S	FF	549.58	29.64	281.19	12.95	302.16	16.4	12.85	2.86
	FS-SS	545.94	49.62	289.88	18.08	328.08	22.4	8.3	1.29
EPHX2 R287Q	RR	547.27	28.7	283.26	11.75	303.39	13.72	9.43	1.14
	RQ-QQ	553.56	58.49	288.56	23.46	348.04	39.51	20.94	10.33
EPHX2 3'UTR	A/A	529.21	32.24	275.66	14.18	290.57	15.69	11.94	2.66
	A/G y G/G	571.82	41.58	294.47	15.65	336.03	22.04	10.53	2.87
EPHX2 K55R	KK	538.4	28.24	283.36	11.9	300.72	14.21	11.88	2.42
	KR-RR	584.06	61.12	287.03	22.63	347.84	32.59	9.36	2.24

Figure S1. Epoxygenase pathway of arachidonic acid metabolism.

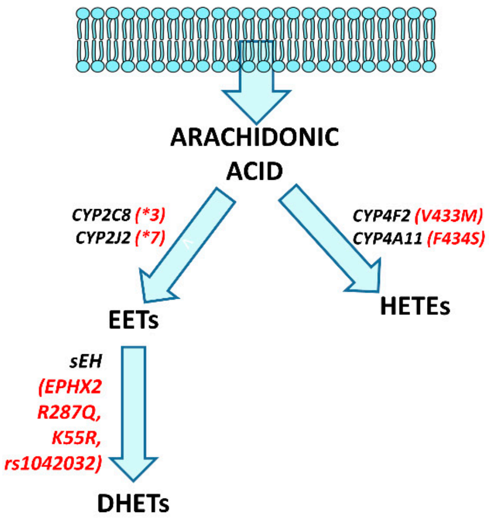

Supplementary Figure S1

Epoxygenase pathway of arachidonic acid metabolism. Genetic polymorphisms determined are indicated in red.

EETs, epoxyeicosatrienoic acids; HETEs, hydroxytetraenoic acids; DHETs, dihydroxyeicosatrienoic acids; sEH, soluble epoxyhydrolase

Figure S2: Cumulative event-free survival for patients with diabetic kidney disease according to the different genotypes considered

Supplementary Figure S2 (I). Cumulative event-free survival for patients with diabetic Kidney disease according to the different genotypes considered

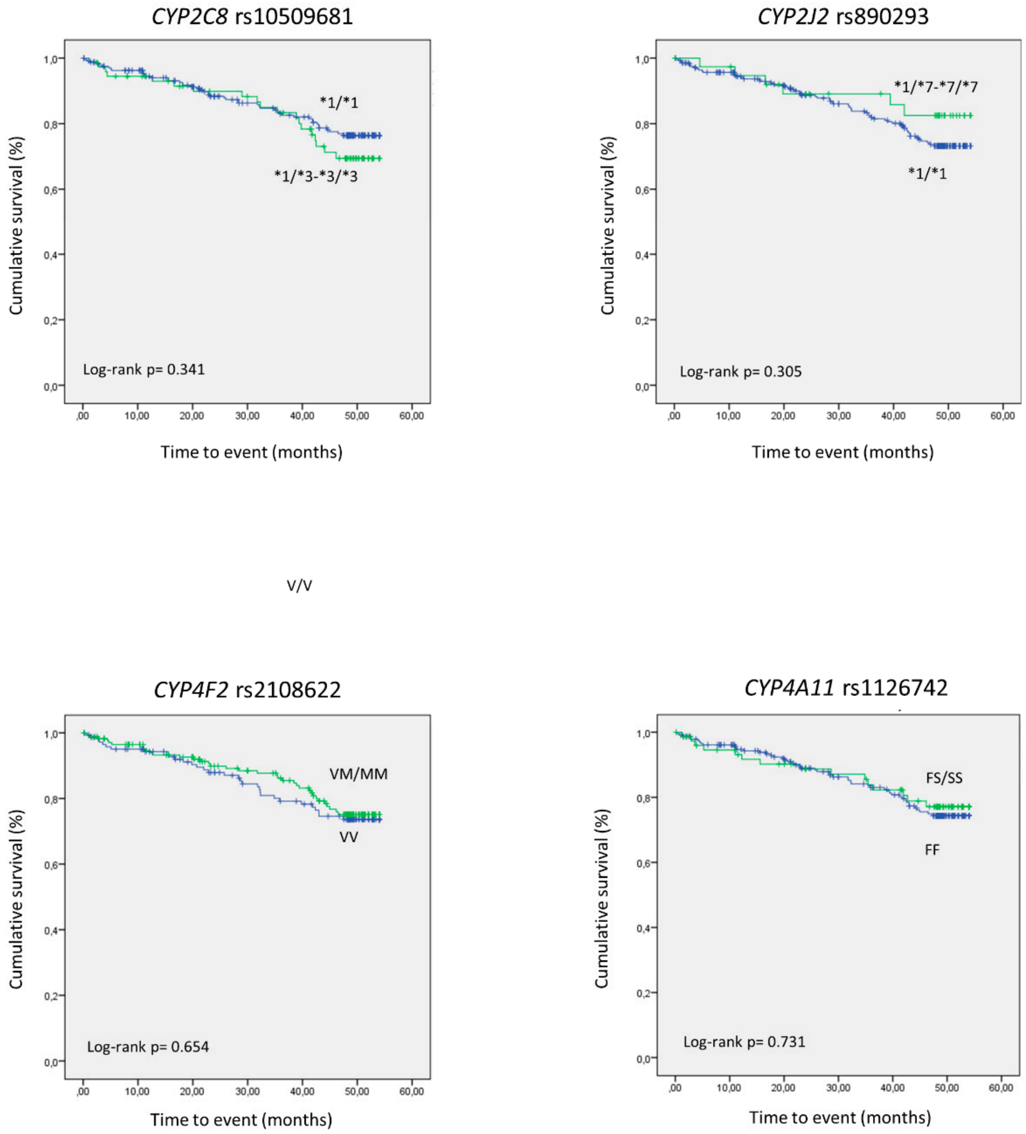

Supplementary Figure S2 (II). Cumulative event-free survival for patients with diabetic Kidney disease according to the different genotypes considered

EPHX2 rs751141

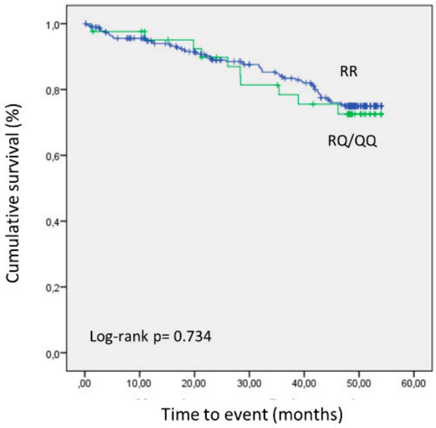

EPHX2 rs1042032

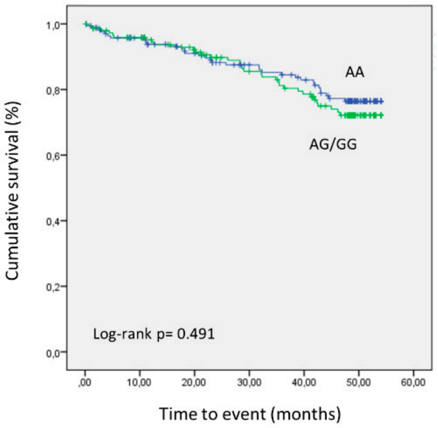

EPHX2 rs41507953

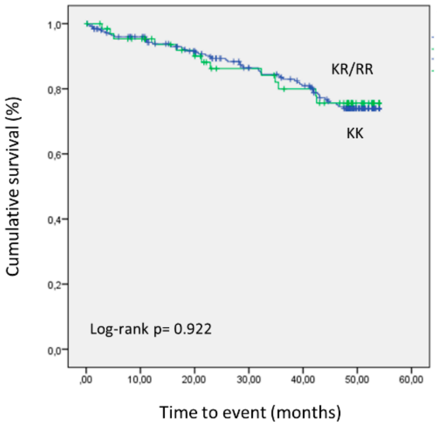

Supplement: Supplementary file 1 [file jcm-10-03980-s001.zip › jcm-1363132-supplementary.pdf]
